# Diagnostic accuracy of transmucosal probe visualization for peri‐implant mucosal phenotype assessment: A cross‐sectional study

**DOI:** 10.1002/jper.70062

**Published:** 2026-01-26

**Authors:** Emilio Couso‐Queiruga, Manrique Fonseca, Diogo Moreira Rodrigues, Gustavo Avila‐Ortiz, Vivianne Chappuis, Clemens Raabe

**Affiliations:** ^1^ Department of Oral Surgery and Stomatology University of Bern School of Dental Medicine Bern Switzerland; ^2^ Department of Reconstructive Dentistry and Gerodontology University of Bern School of Dental Medicine Bern Switzerland; ^3^ Department of Periodontology National Institute of Dental Sciences Niterói, Rio de Janeiro Brazil; ^4^ Department of Periodontics and Oral Medicine University of Michigan School of Dentistry Ann Arbor Michigan USA

**Keywords:** dental implants, esthetics, outcome assessment, outcome measures, phenotype

## Abstract

**Background:**

This study evaluated the diagnostic accuracy of visual assessment of mucosal transparency using a standard periodontal probe (VAT) to differentiate between thin and thick peri‐implant mucosal phenotypes, compared to horizontal transmucosal probing (HTP). A secondary objective was to assess facial mucosal thickness (FMT) threshold values for peri‐implant mucosa phenotype classification.

**Methods:**

Adult subjects with at least one non‐molar single implant‐supported prosthesis (ISP) were screened. To evaluate VAT, a standard periodontal probe was inserted through the peri‐implant sulcus on the midfacial aspect. FMT was measured via HTP using an endodontic spreader.

**Results:**

A total of 247 subjects and 281 ISPs constituted the study population. Setting a 2 mm threshold, 66.2% of sites were classified as thick and 33.8% as thin. Compared to HTP, VAT demonstrated a sensitivity (SE) of 12.6%, a specificity (SP) of 96.2%, a positive predictive value (PPV) of 63%, and a negative predictive value (NPV) of 66%. At the 1.5 mm threshold, 86.1% of sites were thick and 13.9% thin, with 25.6% SE, 96.3% SP, 53% PPV, and 89% NPV. At the 1 mm threshold, 92.2% of sites were thick and 7.8% thin, with 41% SE, 96% SP, 47% PPV, and 95% NPV. Receiver operating characteristic analysis revealed the best‐fitting balance for PPV (58%) and NPV (91%) at an FMT of 1.25 mm.

**Conclusions:**

Regardless of the FMT threshold applied, VAT demonstrated limited diagnostic accuracy, in particular for thin phenotypes, proving unreliable for the dichotomic characterization of thin and thick peri‐implant mucosal phenotypes. This study was approved by the ethical committee for clinical studies in the canton of Bern, Switzerland (KEK‐BE‐No. 2023‐01962).

## Plain language summary

This study primarily aimed to evaluate the diagnostic accuracy of two methods to characterize the thickness of the peri‐implant mucosa: a visual assessment of mucosal transparency of a standard periodontal probe inserted into the peri‐implant sulcus (VAT) and horizontal transmucosal probing using a puncturing instrument (HTP). Adult subjects with at least one implant‐supported prosthesis were included. Regardless of the facial mucosal thickness threshold (2, 1.5, or 1 mm), VAT exhibited limited reliability in accurately identifying thin and thick mucosal phenotypes compared to HTP.

## 1 INTRODUCTION

An accurate assessment of the components of the periodontal and peri‐implant phenotype, including mucosal thickness, keratinized mucosa width (KMW), supracrestal tissue height, and the underlying bone thickness, is essential for clinical decision‐making and evaluating treatment outcomes.^1^, ^2^, ^3^, ^4^ These phenotypic characteristics are site‐specific and can be influenced by various environmental factors, such as inflammatory diseases, trauma, and surgical procedures that involve hard and soft tissue augmentation or resection.^2^, ^5^, ^6^, ^7^, ^8^, ^9^

Numerous qualitative and quantitative methods have been introduced to measure and classify the dimensions of the hard and soft tissues around teeth and osseointegrated dental implants. These methods range from a simple visual inspection of the superficial characteristics of the region of interest^10^ or the visibility through the mucosa of a periodontal probe inserted into the sulcus,^11^, ^12^, ^13^, ^14^, ^15^, ^16^ direct thickness assessments via horizontal transmucosal probing (HTP)^17^, ^18^, ^19^ or using a spring caliper,^12^, ^17^ to advanced imaging modalities such as ultrasonography,^20^, ^21^, ^22^, ^23^ and cone‐beam computed tomography (CBCT),^24^, ^25^ with or without the superimposition of surface scans.^17^, ^26^, ^27^ Among these, one of the most widely adopted non‐invasive methods, which was endorsed by the 2017 World Workshop on the Classification of Periodontal and Peri‐implant Diseases and Conditions, is the visual assessment of mucosal transparency of a standard periodontal probe inserted into the periodontal sulcus (VAT).^28^ However, a recent meta‐analysis that evaluated its diagnostic accuracy around teeth suggests that this method may lead to inaccuracies concerning the differentiation between thin and thick gingival phenotypes.^29^

In implant dentistry, heterogeneity among current methods for peri‐implant phenotype characterization and assessment^20^, ^23^, ^26^, ^30^, ^31^, ^32^, ^33^, ^34^, ^35^ may limit external validity and reduce the comparability of therapeutic outcomes across clinical studies. Additionally, evidence is scarce regarding the reliability of simple, non‐invasive qualitative assessment methods, such as VAT, to accurately distinguish between thin and thick peri‐implant mucosal phenotypes. Similarly, there is still no consensus on a specific threshold to distinguish between thin and thick mucosal phenotypes that would ensure predictable long‐term functional and esthetic outcomes and minimize marginal bone loss and mucosal recession. Although a 2 mm threshold has been suggested due to its clinical implications, further evaluation of different thresholds is still needed.^1^ Therefore, the primary aim of this cross‐sectional study was to evaluate the diagnostic accuracy of VAT compared with HTP for classifying peri‐implant mucosal phenotypes. Secondary objectives included determining optimal threshold values for peri‐implant mucosal thickness classification and investigating associations with other phenotypical variables.

## 2 MATERIALS AND METHODS

### 2.1 Study design, ethical approval, and setting

This study was designed as a single‐center cross‐sectional clinical investigation and adhered to the Strengthening the Reporting of Observational Studies in Epidemiology (STROBE) guidelines^36^ and the Standards for Reporting Diagnostic Accuracy (STARD).^37^ The research protocol received approval from the ethical committee for clinical studies in the canton of Bern, Switzerland (KEK‐BE‐No. 2023‐01962) and was conducted in accordance with the Declaration of Helsinki. Data were collected at the Department of Oral Surgery and Stomatology, School of Dental Medicine, University of Bern, Switzerland, between November 2023 and September 2024.

### 2.2 Recruitment

Adult subjects with non‐molar, single implant‐supported prostheses (ISP) with adjacent teeth in need of a comprehensive dental evaluation were eligible to participate in this study. Before inclusion, all potential participants were required to read, comprehend, and sign an informed consent form detailing the study's objectives and methodology. The inclusion criteria were: (1) ≥18 years old; (2) the presence of at least 1 bone‐ or tissue‐level ISP in a non‐molar single edentulous site in the maxilla or mandible, placed in the Department of Oral Surgery and Stomatology at the University of Bern. The exclusion criteria were: (1) diagnosis or history of peri‐implantitis;^38^, ^39^ (2) presence of an abnormal mucosal color (e.g., metal tattoo or congenital pigmentation); (3) implant malposition; (4) any disabilities or barriers that may hinder comprehension, reading, or signing of the informed consent.

### 2.3 Clinical procedures

**FIGURE 1:**
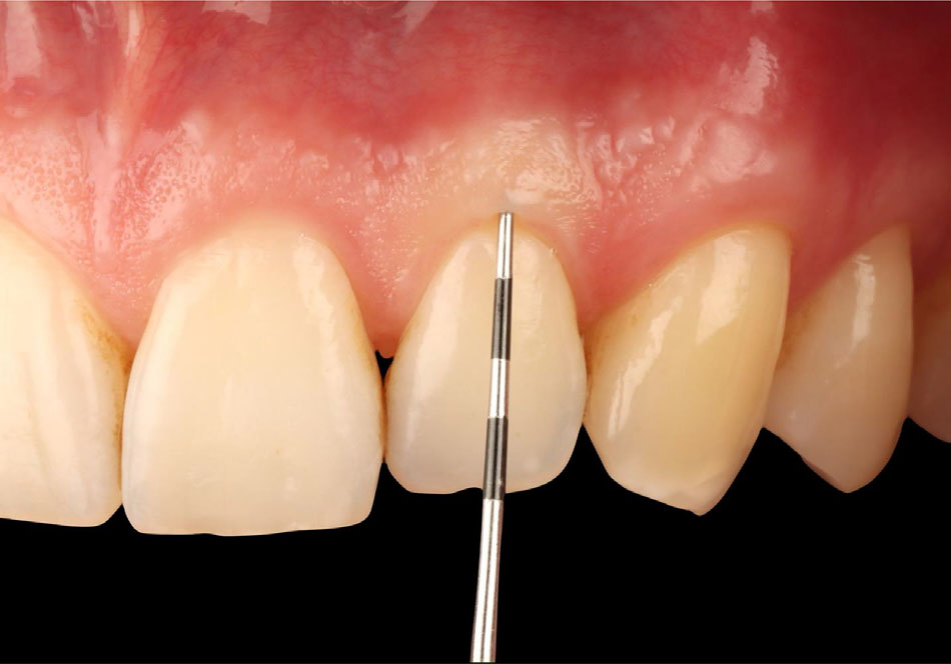
Insertion of a standard periodontal probe into the peri‐implant sulcus to visually assess and classify the mucosal phenotype as thin or thick based on probe visibility.

**FIGURE 2:**
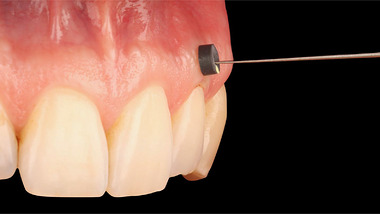
Use of an endodontic spreader to measure mucosal thickness 3 mm apical to the mucosal margin.

**FIGURE 3:**
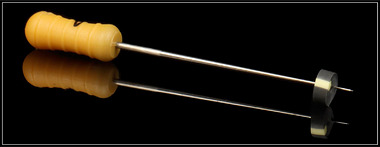
Illustration of an endodontic spreader with the circular rubber stopper positioned at a peripheral point, distant from the pre‐made orifice, to prevent any unintended movement of the stopper.

All clinical procedures conducted during the comprehensive oral evaluation were performed by two calibrated examiners (E.C‐Q. and C.R.). The calibration process involved a discussion meeting to review the study protocol and a preliminary joint assessment of 10 randomly selected sites, ensuring consistency and standardization when using both assessment methods (VAT and HTP). Clinical measurements were made using a periodontal probe,* including probing depth (PDs), bleeding on probing (BOP), and suppuration on probing (SOP) at six sites around the dental implant and adjacent teeth: mesio‐facial, mid‐facial, disto‐facial, mesio‐lingual, mid‐lingual, and disto‐lingual. Additionally, KMW was measured at the mid‐facial aspect as the distance from the mucosal margin to the mucogingival junction in millimeters. The visibility through the mucosa of a periodontal probe inserted into the sulcus at the mid‐facial was recorded as yes/no, as displayed in Figure 1, corresponding with the index test. Similarly, for the reference standard, facial mucosal thickness (FMT) was directly measured using the HTP method with a standard no. 20 endodontic finger spreader† at 3 mm apical to the mucosal margin, and perpendicular to the long axis of the ISP, as illustrated in Figure 2. The tip of the endodontic spreader was carefully inserted through the circular rubber stopper at a peripheral point, away from the pre‐made orifice, to prevent unwanted movement of the stopper and reduce assessment errors, as depicted in Figure 3 and previously reported.^17^ Upon encountering tactile resistance, the rubber stopper was gently placed over the mucosal surface. Consequently, the distance between the tip of the endodontic spreader and the internal edge of the rubber stopper was measured with a stainless‐steel ruler.

Finally, a periapical radiograph was taken centered on the region of interest for diagnostic evaluation. Patient demographic and dental history data, including age, sex, implant type (i.e., bone‐ or tissue‐level) dimensions (i.e., diameter and length), platform, and type of ISP (cement‐ or screw‐retained) were also recorded.

### 2.4 Statistical analysis

All data analyses were conducted using SPSS software.‡ The normality of data distribution was assessed using the Shapiro‐Wilk test. Descriptive statistics were presented as means and standard deviations (SDs) for continuous variables, while categorical variables were expressed as frequencies and percentages. The chi‐squared test was applied to evaluate the association between peri‐implant mucosal phenotype classifications obtained through both assessment methods. Comparisons of mean values for FMT, KMW, and PD were performed using the Student's *t*‐test. The correlation between FMT, KMW, and PD was analyzed using Pearson's correlation coefficient.

The diagnostic accuracy of VAT as the index test was evaluated against the HTP, which served as the reference standard, given that HTP provides a direct and quantitative assessment of FMT. Sensitivity (SE) (defined as the ability to correctly classify the thin phenotype), specificity (SP) (the ability to classify the thick phenotype), accuracy, positive predictive value (PPV), and negative predictive value (NPV), alongside the area under the curve (AUC), and 95% confidence intervals, were computed for VAT. A receiver operating characteristics curve (ROC) curve was generated to identify the FMT cutoff, considering Youden's index, which optimally discriminates between thin and thick mucosal phenotypes. In this case, the continuous value was the FMT, and the dichotomous value was the VAT outcome, so the SE and SP refer to the FMT's ability to predict the transparency of the mucosa. This resulted in a reversed contingency table, meaning that, when the VAT outcomes are treated as a diagnostic test, the SE of the “reversed” test corresponds to the PPV, while the SP corresponds to the NPV.^40^ Based on the HTP (reference standard), sites were categorized into thin and thick phenotypes using the different thresholds of 1.0, 1.5, and 2.0 mm, and subsequently compared to the VAT results.

Inter‐rater reliability for clinical measurements was assessed using intraclass correlation coefficients (ICC).^41^ A significance level of *α* = 0.05 was applied for all statistical tests.

### 2.5 Sample size calculation

To determine the appropriate sample size for detecting statistically significant differences in phenotype assessment, a posteriori assessment was conducted on the first 50 ISPs. This pilot revealed proportions of 10% (thin phenotype) and 90% (thick phenotype) according to the VAT method. Based on these preliminary findings, a sample size calculation targeting 99% statistical power and a 5% margin of error yielded an estimated required sample size of 239 ISPs.

## 3 RESULTS

### 3.1 Study population and sample characteristics

**FIGURE 4:**
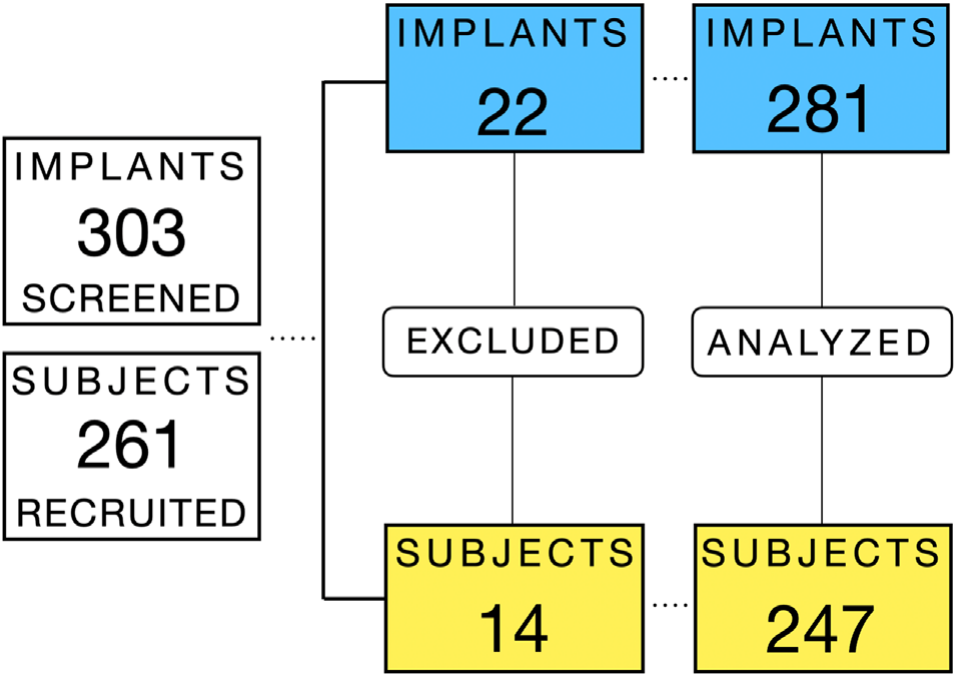
Flowchart illustrating included subjects and implants.

A total of 261 subjects with 303 ISPs were initially screened. However, 13 ISPs were excluded due to peri‐implantitis, two ISPs were not present at the time of evaluation due to previous explanation, and seven could not be assessed due to the patient's refusal to participate in the study. Consequently, a total of 247 subjects with 281 non‐molar ISPs were included in this study, as shown in Figure 4. The study population consisted of 138 females (55.9%) and 109 males (44.1%) with a mean age of 59.7 ± 16.3 years. Regarding ISP distribution, 223 (79.3%) were located in the maxilla and 58 (20.7%) in the mandible. Of these, 69 were located in the central incisors, 64 in the lateral incisors, 19 in the canines, 58 in the first premolars, and 71 in the second premolars. Implant types involved 166 bone‐level implants and 115 tissue‐level implants, with diameters of 3.3 mm (*n* = 137) and 4.1 mm (*n* = 144), and lengths of 8 mm (*n* = 21), 10 mm (*n* = 138), 12 mm (*n* = 107), and 14 mm (*n* = 15). Implant platforms were categorized as NC (*n* = 77), NNC (*n* = 59), RC (*n* = 89), and RN (*n* = 56). Implants were in function for a mean follow‐up of 11.3 ± 1.5 months.

Forty‐five (20.2%) ISPs were cement‐retained, while 178 (79.8%) were screw‐retained. Mean PD was 4.3 ± 1.4 mm. A total of 42 ISPs exhibited no BOP, while 34 ISPs presented BOP at a single site. Additionally, 14 ISPs displayed SOP, with 6 of these cases limited to a single site. Mean KMW was 3.7 ± 1.8 mm, ranging from 0 to 9 mm. Mean FMT was 2.2 ± 0.8 mm, ranging from 0.5 to 5 mm.

Apical migration of the mucosal margin/recession was observed in 228 ISPs (81.1%). However, clinical exposure of the prosthetic abutment/implant shoulder was noted in only 28 of them (12.3%). Finally, 28.1% and 71.9% of ISPs were diagnosed with peri‐implant health and peri‐implant mucositis, respectively.

### 3.2 Intra‐examiner reliability

The ICC for all clinical parameters evaluated between examiners was 0.95, indicating excellent inter‐rater reliability and agreement across all clinical study parameters.

### 3.3 Diagnostic accuracy of VAT

For HTP and applying a mucosal phenotype threshold of 2 mm (thick: ≥2 mm; thin: <2 mm), 186 sites (66.2%) were classified as thick, while 95 sites (33.8%) were classified as thin. Using the VAT method, 262 (93.2%) sites exhibited a thick phenotype. Of these, 179 (68.3%) were also classified as thick using HTP, whereas 83 (31.7%) were classified as thin. Conversely, a thin phenotype was diagnosed using the VAT method in only 19 sites (6.8%). Among these, 7 (36.8%) were identified as thick and 12 (63.2%) as thin using the HTP, as shown in Table 1. The SE and SP of VAT versus HTP were 12.6% and 96.2%, respectively. The PPV and NPV were 63.1% and 66.1%, respectively.

**TABLE 1:** Comparison of mucosal phenotype classification according to the facial mucosal thickness and assessment method

Threshold and method of assessment	Thick phenotype (*n*, %)	Thin phenotype (*n*, %)	Total (*n*, %)
Threshold: 2 mm			
Horizontal transmucosal probing	186 (66.2%)	95 (33.8%)	281 (100%)
Periodontal probe visibility through the mucosa	262 (93.2%)	19 (6.8%)	281 (100%)
Threshold: 1.5 mm			
Horizontal transmucosal probing	242 (86.1%)	39 (13.9%)	281 (100%)
Periodontal probe visibility through the mucosa	262 (93.2%)	19 (6.8%)	281 (100%)
Threshold: 1 mm			
Horizontal transmucosal probing	259 (92.3%)	22 (7.8%)	281 (100%)
Periodontal probe visibility through the mucosa	262 (93.2%)	19 (6.8%)	281 (100%)

When the threshold was set at 1.5 mm (thick: ≥1.5 mm; thin: <1.5 mm), 242 sites (86.1%) were classified as thick, while 39 sites (13.9%) were classified as thin using HTP. Using the VAT method, 262 (93.2%) sites were diagnosed as thick. Among these, 232 (88.5%) were confirmed as thick using HTP, whereas 30 (11.5%) were classified as thin. Similarly, a thin phenotype was diagnosed using VAT in only 19 sites (6.8%). Among these, 10 (52.6%) were classified as thick and 9 (47.4%) as thin using HTP, as displayed in Table 1. Therefore, the SE and SP of VAT versus HTP were 25.6% and 96.3%, respectively. The PPV and NPV were 52.6% and 88.9%, respectively.

When the threshold was set at 1 mm (thick: ≥1 mm; thin: <1 mm), 259 sites (92.2%) were classified as thick, while 22 sites (7.8%) were classified as thin using HTP. Using the VAT method, 262 (93.2%) sites were diagnosed as thick. Among these, 249 (88.6%) were confirmed as thick using HTP, whereas 13 (4.6%) were classified as thin. Similarly, a thin phenotype was diagnosed using VAT in only 19 sites (6.8%). Among these, 10 (52.6%) were classified as thick and 9 (47.4%) as thin using HTP, as displayed in Table 1. VAT was associated with HTP (chi‐squared = 44.1; *p* < 0.001). Therefore, the SE and SP of VAT versus HTP were 41%, and 96%, respectively. The PPV and NPV were 47.3% and 95%, respectively.

### 3.4 Comparison of mean measurements of the phenotypical variables according to the methods of assessment

The stratification of mucosal phenotype by VAT showed that KMW was narrower (thin: 2.5 ± 1.6 mm; thick: 3.8 ± 1.9 mm), and FMT was thinner (thin: 1.4 ± 0.7 mm; thick: 2.2 ± 0.8 mm) in sites exhibiting mucosal transparency. However, no differences were observed regarding PDs (thin: 3.4 ± 0.9 mm; thick: 3.8 ± 1.1 mm), as shown in Table 2.

**TABLE 2:** Comparison of keratinized mucosa width, facial mucosal thickness, and facial probing depth values, in sites classified as thin and thick mucosal phenotype, according to the assessment method and thresholds.

	Keratinized mucosa width			Facial mucosal thickness			Facial probing depth		
Assessment method	Thin mean (SD)	Thick mean (SD)	*p*‐value	Thin mean (SD)	Thick mean (SD)	*p*‐value	Thin mean (SD)	Thick mean (SD)	*p*‐value
Periodontal probe visibility through the mucosa	2.5 (1.6)	3.8 (1.9)	0.158	1.4 (0.7)	2.2 (0.8)	0.003^*^	3.4 (0.9)	3.8 (1.1)	<0.001^*^
Horizontal transmucosal probing and threshold set at 2 mm	3.0 (1.6)	4.1 (1.8)	<0.001^*^	1.4 (0.33)	2.6 (0.6)	<0.001^*^	3.3 (0.9)	3.9 (1.1)	<0.001^*^
Horizontal transmucosal probing and threshold set at 1.5 mm	2.4 (1.4)	3.9 (1.8)	<0.001^*^	1.0 (0.2)	2.4 (0.7)	<0.001^*^	3.2 (0.8)	3.8 (1.1)	<0.001^*^
Horizontal transmucosal probing and threshold set at 1 mm	2.3 (1.2)	3.8 (1.8)	<0.001^*^	0.9 (0.2)	2.3 (0.7)	<0.001^*^	2.9 (0.8)	3.8 (1)	<0.001^*^

* Indicates statistical significance (*p* < 0.005).

When the mucosal phenotype was assessed using HTP with thresholds of 2 mm, 1.5 mm, and 1 mm to classify thin and thick phenotypes, the Student's *t*‐test indicated significant differences with KMW (*p* < 0.001), FMT (*p* < 0.001), and PDs (*p* < 0.001). In particular, thicker mucosal phenotypes were associated with higher PD values, wider KMW, and greater FMT compared to thinner phenotypes, as depicted in Table 2.

When stratifying the mucosal phenotype using a threshold of 2 mm, mean KMW values were 3.0 ± 1.6 mm for the thin group and 4.1 ± 1.8 mm for the thick group. Mean FMT values were 1.4 ± 0.3 mm and 2.6 ± 0.6 mm, respectively, for the thin and thick phenotypes, while mean PD values were 3.3 ± 0.9 mm for the thin phenotype and 3.9 ± 1.1 mm for the thick phenotype.

Similarly, when stratifying the mucosal phenotype using a threshold of 1.5 mm, mean KMW values were 2.4 ± 1.4 mm for the thin group and 3.9 ± 1.8 mm for the thick group. Mean FMT values were 1.0 ± 0.2 mm for the thin phenotype and 2.4 ± 0.7 mm for the thick phenotype, while mean PD values were 3.2 ± 0.8 mm for the thin phenotype and 3.8 ± 1.1 mm for the thick phenotype.

Similarly, when stratifying the mucosal phenotype using a threshold of 1 mm, mean KMW values were 2.3 ± 1.2 mm for the thin group and 3.8 ± 1.8 mm for the thick group, respectively. Mean FMT values were 0.9 ± 0.2 mm for the thin phenotype and 2.3 ± 0.7 mm for the thick phenotype, while mean PD values were 2.9 ± 0.8 mm for the thin phenotype and 3.8 ± 1 mm for the thick phenotype.

Finally, FMT was positively correlated with facial PD (Pearson's *r* = 0.314, *p* < 0.001), and KMW (Pearson's *r* = 0.317, *p* < 0.001). Additionally, KMW was also positively correlated with facial PD (Pearson's *r* = 0.295), *p* < 0.001).

### 3.5 Correlation analysis of phenotypical variables and patient‐ and implant‐related variables

Compared to females, males exhibited greater KMW (3.9 ± 1.8 mm vs. 3.5 ± 1.9 mm; *p *= 0.122), significantly thicker FMT (2.3 ± 0.8 mm vs. 2.1 ± 0.8 mm; *p* = 0.019), and deeper mean PD (3.9 ± 1.1 mm vs. 3.6 ± 1.1 mm; *p* = 0.046). Regarding ISP location, mandibular sites showed significantly narrower KMW (2.8 ± 0.8 mm vs. 3.9 ± 0.7 mm; *p* < 0.001) and shallower PD (3.4 ± 0.7 mm vs. 3.8 ± 0.2 mm; *p* < 0.001) compared to maxillary sites, while FMT values were comparable between jaws (2.1 ± 0.7 mm vs. 2.2 ± 0.3 mm; *p* = 0.165). Lastly, bone‐level implant sites demonstrated significantly greater KMW (3.8 ± 1.1 mm vs. 3.4 ± 0.9 mm; *p* < 0.001) compared to tissue‐level implants, with no significant differences observed regarding FMT (3.9 ± 1.1 mm vs. 3.3 ± 0.9 mm;* p *= 0.113) or PD mean values (3.8 ± 1.1 mm vs. 3.2 ± 0.9 mm; *p* = 0.122). Table S1 (in the online *Journal of* *Periodontology*) presents the differences in clinical parameters according to sex, ISP location, and implant type.

### 3.6 VAT performance according to facial mucosal thickness

**FIGURE 5:**
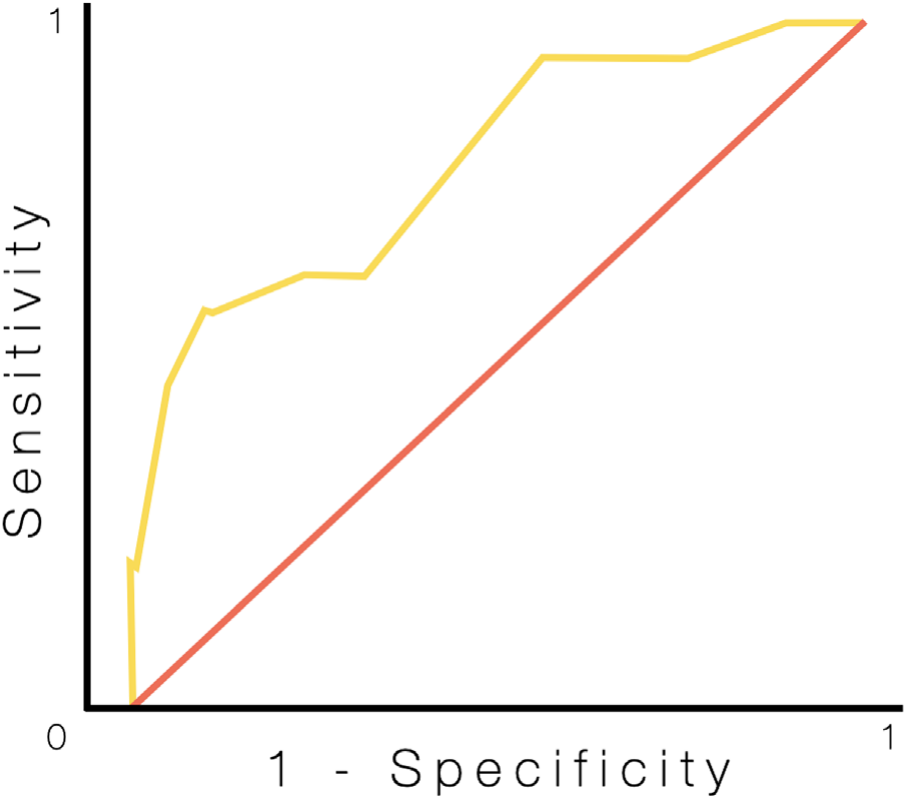
The receiver operating characteristic (ROC) curve shows the diagnostic performance of the transmucosal probe visibility related to the facial peri‐implant mucosal thickness.

At a cutoff value of 1.25 mm for FMT, VAT demonstrated good diagnostic accuracy, with an AUC of 0.794. At this threshold, the NPV was high (91%), indicating a strong ability to correctly identify thick mucosa, while the PPV was moderate (58%), reflecting a more limited capacity to detect thin mucosa. The corresponding Youden's index of 0.480 suggests a reasonable, though not optimal, balance between SE and SP (Table S2 in the online *Journal of Periodontology*). Figure 5 illustrates the ROC curve for the diagnostic performance of VAT in relation to mucosal thickness.

## 4 DISCUSSION

This single‐center cross‐sectional study aimed at evaluating the diagnostic accuracy of VAT compared with HTP for classifying peri‐implant mucosal phenotypes. To the best of our knowledge, this is the first clinical study comparing VAT with HTP. The main finding of this study is that VAT is an unreliable method for distinguishing between thin and thick peri‐implant mucosal phenotypes. From a clinical perspective, although VAT is practical, clinicians should exercise caution when relying on it as the sole diagnostic tool. Additional objective methods should be incorporated to minimize the risk of misclassification and to support more accurate clinical decision‐making.

It was also observed that FMT thresholds of 2, 1.5, and 1 mm to differentiate between thin and thick phenotypes did not significantly affect the reliability of the VAT method. However, FMT values tended to be lower in sites where mucosal transparency was visually observed. Interestingly, we found that thicker FMT was associated with wider KMW and greater mean PD values. Conversely, thinner FMT was correlated with narrower KMW and lower mean PD values. Additionally, males exhibited wider KMW, thicker FMT, and greater PD mean values compared to females. ISPs located in the mandible generally exhibited narrower KMW than those in the maxilla. In contrast, no significant differences were observed between bone‐ and tissue‐level implants in terms of phenotypical features of the peri‐implant mucosa or other clinical parameters.

Various thresholds have been proposed in the literature to define mucosal phenotypes, with the classification of thin and thick phenotypes largely depending on the selected landmarks and the cutoff values used.^42^ In the present study, when applying a clinically relevant threshold of 2 mm,^1^, ^43^, ^44^, ^45^ the VAT method demonstrated very low SE but maintained high SP in comparison to the HTP method. The likelihood of correctly identifying positive cases was moderate, and the ability to rule out negative cases was similarly moderate. Notably, lowering the threshold to 1.5 and 1 mm led to an improvement in SE, while SP remained largely unchanged. This adjustment reduced the accuracy of positive predictions but significantly enhanced the accuracy of negative ones. These findings align with those from a recently published meta‐analysis, which also found that the diagnostic performance of the VAT method in tooth‐sites is strongly influenced by the FMT threshold values and the anatomical landmark (e.g., apico‐coronal level) where the gingival thickness was obtained. Especially, the meta‐analysis observed a mean gingival thickness of approximately 1.4 mm at 3 mm apical to the gingival margin, compared to 2.2 mm reported in the present study.^14^ Despite the adoption of the 2 mm threshold according to previous studies^44^, ^45^, ^46^ and the classification suggested by Avila‐Ortiz and collaborators,^1^ we also utilized the 1.5 mm and 1 mm cutoffs to classify thin and thick phenotypes. At any rate, our data indicate that relying solely on VAT may lead to gross misclassifications of the mucosal phenotype.

When potential associations of local phenotypical variables with the method of assessment were evaluated, we found that both KMW and FMT were significantly, but distinctly correlated in sites presenting a thin or thick phenotype determined by using the VAT method. Specifically, a narrower KMW and a thinner FMT were associated with an increased likelihood of observing the gray shade of the periodontal probe through the mucosa when non‐invasively inserted into the peri‐implant sulcus. These findings are consistent with those observed by other investigators who reported that thinner FMT increases the probability of a positive visualization of the probe using VAT.^11^, ^14^, ^47^, ^48^ However, it is important to note that, despite this correlation, visual assessment may lead to inaccuracies in determining the soft‐tissue phenotype, regardless of the FMT.^14^, ^49^, ^50^ Similarly, when the sites were stratified according to FMT, to differentiate between thin and thick, it was observed that the thinner the FMT, the narrower the KMW, and the lower the PD. Contrarily, the thicker the FMT, the wider the KMW, and the greater the PD. However, VAT was also visually assessed as positive in some sites presenting a thick mucosal phenotype according to HTP. In these situations, the mucosa was often more stretched than under normal conditions during probe insertion through the sulcus. This was likely influenced by the prosthetic design and the quality of the mucosal seal around the transmucosal prosthetic components, particularly in the presence of mobile mucosa. This phenomenon can be explained by previous findings from ex vivo porcine and clinical studies that demonstrated a greater stretchability of the alveolar mucosa as compared to the gingiva.^51^, ^52^, ^53^

Regarding sex‐related differences, males generally exhibited wider KMW, thicker FMT, and greater PD values. These findings are consistent with previous studies that characterized the periodontal phenotype^29^, ^54^, ^55^ but contrast with those of a retrospective study that found no differences between KMW and sex, patient age, or PDs around ISPs. When analyzing ISP location and its relationship with local phenotypical characteristics, mandibular sites exhibited significantly narrower KMW compared to the maxilla. However, no significant differences were noted regarding FMT. This pattern aligns with findings reported for natural teeth in classic studies^56^, ^57^ and a recent meta‐analysis,^58^ as well as a retrospective study involving dental implants.^59^ Additionally, bone‐level implants were associated with wider KMW, although this difference does not hold clinical significance. Furthermore, no significant differences in FMT or PD values were observed when compared to tissue‐level implants. In summary, these results suggest that the type of implant does not significantly affect the assessment of the mucosal phenotype when classifying it as thin or thick using either VAT or HTP.

ROC analysis revealed that at a cutoff of 1.25 mm of FMT, VAT exhibited a high NPV of 91%, indicating that this method of assessment is highly reliable with FMT values at or above this threshold, indicating a thick phenotype. However, the observed PPV of 58% reflects a limited diagnostic performance to consistently identify FMT below 1.25 mm, potentially leading to underdiagnosis of a thin mucosal phenotype. The corresponding Youden's Index of 0.480 indicated a fair trade‐off between SE and SP, though not optimal. These findings support the clinical usefulness of VAT as a simple, non‐invasive assessment method for identifying thicker mucosal phenotypes, but also highlight its limitations in identifying thinner FMT. To the best of our knowledge, this is the first study to evaluate the accuracy of VAT in relation to FMT around dental implants. Interestingly, previous studies assessing the periodontal phenotype using VAT have consistently reported the highest Youden's index values at a gingival thickness of 0.8 mm. This threshold was used to differentiate between thin and thick gingival phenotypes, utilizing standard or colored periodontal probes.^18^, ^40^, ^42^, ^47^ The differences with the findings from this study may be attributed to the distinct characteristics of the soft tissue surrounding teeth versus dental implants.^60^, ^61^

This cross‐sectional study has several limitations. First, with the purpose of homogenizing the sample, only non‐molar ISPs were included, and sites presenting periimplantitis were excluded, but this limits the generalizability of the findings. Second, VAT and FMT were performed only once per calibrated examiner, preventing an assessment of intra‐observer reliability. However, both examiners underwent calibration before data collection, and none of the patients received local anesthesia when the HTP method was used to avoid changing the native features of the mucosa, minimizing potential variability. Third, the findings may not apply to posterior sites, where HTP can be challenging due to anatomical factors and the difficulty of perpendicularly assessing FMT. Fourth, other variables such as platform height, emergence profile, and or implant depth were not evaluated. Finally, the study did not compare FMT measurements at different apico‐coronal levels or with other assessment methods, such as ultrasonography or the use of CBCT with or without the superimposition of surface scans. Nevertheless, it is important to highlight that recent studies have shown that HTP has a high diagnostic odds ratio (40.75) to discriminate between thin and thick gingival phenotypes,^14^ and a strong association with gingival thickness determined in histologic samples from fresh frozen cadavers.^19^ Future research in the context of the peri‐implant phenotype evaluation should aim to evaluate the accuracy and precision of both invasive and non‐invasive assessment techniques through direct comparison with clinical measurements. In addition, the most suitable spot for thickness measurements must be discussed for standardization in future studies.

## 5 CONCLUSIONS

VAT is an unreliable method for distinguishing between thin and thick peri‐implant mucosal phenotypes. This non‐invasive assessment method does not provide results comparable to direct transmucosal probing and may lead to inaccurate diagnoses.

## AUTHOR CONTRIBUTIONS

E.C‐Q. conceived and designed the project. E.C‐Q., M.F., and C.R. contributed to data acquisition. E.C‐Q., D.M.R., and G.A‐O. participated in data analysis and interpretation. E.C‐Q. led the writing, while M.F., D.M.R., G.A‐O., V.C., and C.R. critically revised the manuscript. All authors provided final approval and agreed to be accountable for all aspects of the scientific work.

## CONFLICT OF INTEREST STATEMENT

The authors have no conflicts of interest to report on this cross‐sectional study's conduct. No financial support or sponsorship was received.

## Supporting information



Supporting Information

## Data Availability

Data are available from the corresponding author upon reasonable request but are not publicly accessible owing to privacy and ethical restrictions.

## Footnotes

* Marquis probe; Hu‐Friedy, Chicago, IL, USA

† Kerr, Kloten, Switzerland

‡ Version 30.0; IBM, Chicago, IL, USA
