# Supplementary material for: Diagnostic accuracy of transmucosal probe visualization for peri‐implant mucosal phenotype assessment: A cross‐sectional study
Source: J Periodontol. 2026 Jan 26;97(7):1407–17. doi: 10.1002/jper.70062 (PMC13380343; doi:10.1002/jper.70062)
Supplement: Supplementary file 1 — Supporting Information [file JPER-97-1407-s001.docx]

**Supplementary Table**

**Table S1.** Comparison of keratinized mucosa width, facial mucosal thickness, and facial probing depth values, according to sex, implant location, and implant type.

**Table S2.** Coordinates the receiver operating characteristic (ROC) curve for horizontal transmucosal probing across varying threshold values to determine the optimal cut-off point for mucosal transparency diagnostic performance.

| Parameter |  | Sex | | | Location | | | Type of implant | | |
| --- | --- | --- | --- | --- | --- | --- | --- | --- | --- | --- |
|  | Overall  Mean  (SD) | Females  Mean (SD) | Males  Mean (SD) | p-value | Maxillae  Mean (SD) | Mandible  Mean (SD) | p-value | Bone-Level  Mean (SD) | Tissue-Level  Mean (SD) | p-value |
| Keratinized mucosa width | 3.7  (1.8) | 3.5  (1.9) | 3.9  (1.8) | 0.122 | 3.9  (0.7) | 2.8  (0.8) | < 0.001* | 3.4  (0.9) | 3.8  (1.1) | < 0.001* |
| Facial Mucosal Thickness | 2.2  (0.8) | 2.1  (0.8) | 2.3  (0.8) | 0.019* | 2.2 (0.3) | 2.1  (0.7) | 0.165 | 3.3  (0.9) | 3.9  (1.1) | 0.113 |
| Facial Probing Depth | 3.8  (1.1) | 3.6  (1.1) | 3.9  (1.1) | 0.046* | 3.8  (0.2) | 3.4  (0.7) | < 0.005* | 3.2  (0.88) | 3.8  (1.1) | 0.122 |
|  |  |  |  |  |  |  |  |  |  |  |
|  |  |  |  |  |  |  |  |  |  |  |

**Table S1.** Comparison of keratinized mucosa width, facial mucosal thickness, and facial probing depth values, according to sex, implant location, and implant type.

| Coordinates of the ROC Curve | | | |
| --- | --- | --- | --- |
| Test Result Variable(s): Horizontal transmucosal probing | | | |
| Positive if Less Than or Equal To | Sensitivity | 1 - Specificity | Youden's Index |
| -.500 | .000 | .000 | .000 |
| .600 | .158 | .000 | .158 |
| .750 | .211 | .000 | .211 |
| .900 | .211 | .004 | .207 |
| 1.100 | .474 | .050 | .424 |
| 1.250 | .579 | .099 | .480 |
| 1.350 | .579 | .103 | .476 |
| 1.450 | .579 | .111 | .468 |
| 1.550 | .632 | .237 | .395 |
| 1.650 | .632 | .248 | .383 |
| 1.750 | .632 | .252 | .380 |
| 1.850 | .632 | .309 | .322 |
| 1.950 | .632 | .317 | .315 |
| 2.100 | .947 | .561 | .386 |
| 2.350 | .947 | .611 | .337 |
| 2.650 | .947 | .733 | .215 |
| 2.900 | .947 | .756 | .192 |
| 3.050 | 1.000 | .893 | .107 |
| 3.150 | 1.000 | .897 | .103 |
| 3.350 | 1.000 | .920 | .080 |
| 3.750 | 1.000 | .950 | .050 |
| 4.500 | 1.000 | .989 | .011 |
| 6.000 | 1.000 | 1.000 | .000 |

**Table S2.** Coordinates the receiver operating characteristic (ROC) curve for horizontal transmucosal probing across varying threshold values to determine the optimal cut-off point for mucosal transparency diagnostic performance.
